# Persistent Atrial Fibrillation: The Role of Left Atrial Posterior Wall Isolation and Ablation Strategies

**DOI:** 10.3390/jcm10143129

**Published:** 2021-07-15

**Authors:** Riyaz A. Kaba, Aziz Momin, John Camm

**Affiliations:** 1 Cardiovascular Clinical Academic Group, Molecular and Clinical Sciences Institute, St. George’s University of London and St. George’s University Hospitals NHS Foundation Trust, London SW17 0QT, UK; Aziz.Momin@stgeorges.nhs.uk (A.M.); jcamm@sgul.ac.uk (J.C.); 2 Ashford and St. Peter’s Hospitals NHS Foundation Trust, Surrey KT16 0PZ, UK

**Keywords:** persistent atrial fibrillation, posterior wall, hybrid ablation, convergent ablation

## Abstract

Atrial fibrillation (AF) is a global disease with rapidly rising incidence and prevalence. It is associated with a higher risk of stroke, dementia, cognitive decline, sudden and cardiovascular death, heart failure and impairment in quality of life. The disease is a major burden on the healthcare system. Paroxysmal AF is typically managed with medications or endocardial catheter ablation to good effect. However, a large proportion of patients with AF have persistent or long-standing persistent AF, which are more complex forms of the condition and thus more difficult to treat. This is in part due to the progressive electro-anatomical changes that occur with AF persistence and the spread of arrhythmogenic triggers and substrates outside of the pulmonary veins. The posterior wall of the left atrium is a common site for these changes and has become a target of ablation strategies to treat these more resistant forms of AF. In this review, we discuss the role of the posterior left atrial wall in persistent and long-standing persistent AF, the limitations of current endocardial-focused treatment strategies, and future perspectives on hybrid epicardial–endocardial approaches to posterior wall isolation or ablation.

## 1. Introduction

Atrial fibrillation (AF) is the most commonly diagnosed sustained cardiac dysrhythmia and is characterised by rapid and irregular activation of the atria. It is associated with an increased risk of ischemic stroke, heart failure and mortality and can have a substantial impact on quality of life. Atrial fibrillation can be paroxysmal, lasting 7 days or less with or without intervention, or be continuous beyond 7 days (persistent, PersAF) or beyond 12 months (long-standing persistent, LSPersAF) [1]. Permanent AF is the term used for long-standing persistent AF when any attempt to restore sinus rhythm has been abandoned or has proved impossible. As each episode of AF continues, progressive electro-anatomical remodelling occurs that may serve to perpetuate and sustain AF, known as ‘AF begets AF’ [2]. Therefore, it is not surprising that treatment strategies vary in effectiveness depending on the extent and duration of AF.

**Figure 1:**
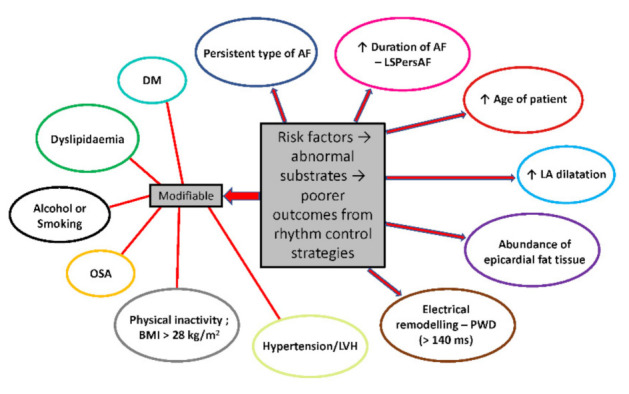
Risk factors for perpetuation of AF. Modifiable risk factors are highlighted separately. LSPersAF, long-standing persistent AF; LA, left atrium; PWD, p-wave duration; LVH, left ventricular hypertrophy; BMI, body mass index; OSA, obstructive sleep apnoea; DM, diabetes mellitus.

Overall, optimal AF management should include a holistic, comprehensive, multidisciplinary approach that collectively considers modifiable risk factors, stroke prevention, and patient- and symptom-focused rate and rhythm control [3] (Figure 1). Using this approach, known as the AF Better Care (ABC) pathway, AF is managed with lifestyle modifications to address risk factors such as obesity and hypertension, and medical therapy which can include anticoagulation for stroke prevention as well as rate and rhythm control drugs depending on the patient and symptoms [1,3]. When antiarrhythmic drugs fail or are intolerable, ablation is recommended. This typically takes form as standalone endocardial catheter ablation or as surgical ablation if performed concomitantly with a primary cardiac surgical procedure. In both cases, pulmonary vein isolation (PVI) is paramount, although other regions often emerge as potential substrates in PersAF [4]. One of these regions, arguably the most influential after the pulmonary veins (PVs), is the posterior wall of the left atrium, which is known to generate AF triggers and is subject to electrical and structural changes that occur with the persistence of AF. However, this region is where endocardial catheter ablation is more limited in its capacity to comprehensively address the AF substrate owing to the elevated risk of collateral damage to adjacent structures such as the oesophagus. This review aims to discuss the published literature on the role of the left atrial posterior wall in PersAF and LSPersAF, outline practical limitations of endocardial catheter ablation to safely and durably isolate the posterior wall and describe the rationale for a hybrid epicardial–endocardial ablation strategy for silencing the PVI and posterior wall.

## 2. Burden of Atrial Fibrillation

Atrial fibrillation was estimated to affect more than 43 million people worldwide in 2016, a figure that continues to rise every year, with observed increases during the last few decades in associated disability and mortality [3]. Atrial fibrillation increases the risk of stroke around five-fold [5], more so with multiple co-existing risk factors, and is also associated with increased mortality [6] even within the first few months of diagnosis [7]. Atrial fibrillation can also overlap with heart failure in that it can exacerbate existing heart failure or lead to tachycardia-induced cardiomyopathy in patients with chronic, poorly managed AF. Therefore, the impact of AF on the global healthcare system is significant. In addition, AF is associated with a decreased quality of life, which can be attributed to the burden of symptoms, as well as the complex interplay with other patient comorbidities commonly associated with AF [8]. In effect, treating the syndrome with AF is not only aimed at reducing the risks of stroke and cardiac death but also decreasing AF burden and, consequently, AF symptoms to improve patient quality of life.

## 3. Treatment of Paroxysmal Atrial Fibrillation

In a seminal 1998 paper, Häissaguerre and colleagues identified the PVs as the primary sites of arrhythmogenicity in paroxysmal AF and that these AF triggers could be destroyed or isolated with radiofrequency ablation [9]. Favourable success rates have been demonstrated for endocardial catheter ablation focused on the PVs for the treatment of drug-refractory paroxysmal AF [10], further supported by advancements in catheter-based radiofrequency, cryoballoon and other technologies [11,12].

## 4. Paroxysmal vs. Persistent Atrial Fibrillation: Differences in Treatment Outcomes

The consistent clinical success of endocardial catheter ablation in paroxysmal AF is not paralleled in persistent and long-standing persistent forms of AF. The discrepancy between paroxysmal and non-paroxysmal AF outcomes is well-evidenced by long-term results of endocardial radiofrequency ablation in these subgroups. With a median follow-up of 4.8 years after circumferential PVI, Ouyang et al. reported 46.6% of 161 patients with paroxysmal AF were free from atrial arrhythmia recurrence after a single procedure, and this success rose to 79.5% with multiple procedures (median 1: range 1–3) [13]. However, in long-standing PersAF, with a median follow-up of 4.7 years, the same investigators reported 20.3% of 202 patients were free from arrhythmia recurrence after PVI with additional CFAE/linear ablation [14]. After multiple procedures (median 2, range 1–5), 45% of patients with LSPersAF were in sinus rhythm. A comprehensive meta-analysis of persistent and long-standing PersAF treatment outcomes reported similarly disappointing results with much shorter follow-up times [15].

**Figure 2:**
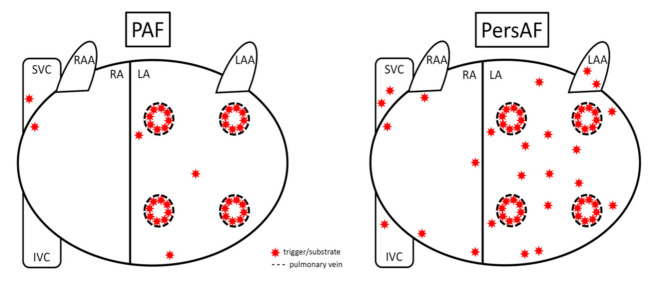
Triggers and substrates for PAF vs. PersAF. In PAF, the majority of these are located within and around the PVs, whereas in PersAF there are many more non-PV locations, especially in the posterior wall (between the four PVs and below the lower PVs). LA, left atrium; LAA, left atrial appendage; RA, right atrium; RAA, right atrial appendage; SVC, superior vena cava; IVC, inferior vena cava.

One explanation for the suboptimal effectiveness of PVI in non-paroxysmal AF is that areas outside of the PVs can drive and act as substrates as AF continues [16]. It has been well-documented that AF triggers are present outside of the PVs [17,18]. While extra-PV triggers may be present in paroxysmal AF, the majority of triggers are located in and around the PVs (Figure 2); this may, at least in part, explain why PV isolation alone is more effective in treating this type of AF [19]. However, as AF becomes persistent, there is a shift towards extra-PV triggers for atrial tachyarrhythmias and, given the progressive electrophysiological and structural changes that occur with the persistence of AF, these extra-PV regions may be appropriate substrates for ablation in PersAF and LSPersAF. Having said that, what and how to ablate in PersAF and LSPersAF is still unclear. Data from the STAR-AF II trial appeared to show that additional endocardial ablation utilising CFAEs or certain linear lesions (roof and mitral lines) adjunctive to PVI did not improve clinical outcomes over PVI alone in PersAF [20]; although, dedicated posterior wall ablation was not specifically tested in this study.

The left atrial posterior wall has been shown to house the highest proportion of non-PV triggers. Lin et al. reported 38% of non-PV ectopic beats emanated from the posterior wall [17]. Additionally, with continued PersAF, the left atrial posterior wall is the most common non-PV site to contain AF re-entrant drivers [16]. In the next section, we review the unique arrhythmogenic properties of the posterior wall that underscore the rationale for its role in PersAF and LSPersAF.

## 5. The Posterior Wall of the Left Atrium in Non-Paroxysmal Atrial Fibrillation

### 5.1. Intrinsic Features

The left atrial posterior wall has several inherent anatomic and electrophysiological properties that are conducive to arrhythmogenicity. When these factors are combined with the structural changes that develop with more prolonged episodes of AF (see next section on ‘Effects of prolonged atrial fibrillation on posterior wall’), the posterior wall then emerges as one of the key regions in the pathophysiology of PersAF. The posterior wall is derived embryonically from the same tissue as the pulmonary veins [21]. Between approximately 6–8 weeks of gestation, the common pulmonary vein, lined with mediastinal myocardium distinct from the primary myocardium that lines systemic venous structures, bifurcates and becomes incorporated into the left atrial wall [22]. Of note, the mediastinal myocardium is composed of fast-conducting cells compared to the slower conducting cells of the primary myocardium. Given the shared tissue origin with the pulmonary veins, it is not surprising then that the posterior left atrial wall is also a site of AF triggers and plays a role in sustaining PersAF.

Myocytes within the left atrial posterior wall have unique electrophysiological properties that may be intrinsically suited to initiate or sustain AF. These cells are characterised by having larger late sodium currents and smaller potassium currents [23]. The intracellular calcium transient and content within the sarcoplasmic reticulum are high. In effect, the cells of the posterior wall have (i) a low resting membrane potential; (ii) short action potential duration; (iii) the shortest refractory period of any cell in the heart. Taken together, these cellular characteristics make the posterior wall prone to misfiring.

Other structural aspects of the posterior wall can contribute to AF initiation and facilitate re-entry. The myocardial fibres in the left atrial posterior wall, particularly near the junction with the pulmonary veins, have a heterogenous orientation with respect to each other [24]. As a consequence, non-uniform anisotropy can occur in which conduction velocity and depolarisation differ between adjacent tissues, including the transition between the epicardial and endocardial layers. Subsequently this can lead to delayed conduction, unidirectional block and, thus, local re-entry.

The autonomic nervous system is a key player in the initiation and sustainment of AF. The posterior wall of the left atrium has the highest density of autonomic neurons in the heart [25]. Ganglionated plexi are groups of autonomic neurons embedded in epicardial fat pads, and some of the ganglionated plexi are located at the posterior left atrium, near the pulmonary veins. Ganglionated plexi are thought to contribute to AF and at times are adjunctive targets in ablation procedures.

### 5.2. Effects of Prolonged Atrial Fibrillation on Posterior Wall

As described above, there are intrinsic functional and anatomical characteristics of the left atrial posterior wall that make it prone to the initiation and maintenance of AF. Once AF occurs and persists over time, progressive changes in the left atrium then serve to propagate and further sustain AF. As such, the left atrial posterior wall is acknowledged as a key AF substrate in persistent forms of the disease. The development of fibrosis is thought to be a contributing factor to the propagation and persistence of AF. Fibrosis can develop due to other cardiac abnormalities or health conditions that are coincident with AF, as well as aging. Fibroblasts comprise 50–70% of cardiac cells [25], and their function is to compose and dynamically maintain the heart’s scaffold [26]. These fibroblasts can differentiate into myofibroblasts under various pathologic conditions, including inflammation and mechanical overload. Myofibroblasts, in turn, produce, turn over and deposit collagen and other extracellular matrix components, which lead to the hardening and scarring of cardiac tissue. This fibrotic tissue can slow conduction, serve as a unidirectional block and contribute to macro re-entry [27]. Cochet et al. demonstrated through MRI delayed enhancement that fibrosis tends to develop on the posterior left atrium [28]. This may be in part due to chronic, increased stress in the regions adjacent to the pericardial reflections that anchor the posterior heart to the chest wall [29]. Additionally, increased pressure and dilation of the left atrium due to prolonged AF leads to stretching, followed by inflammation and leading to fibrosis [30]. 

The accumulation of epicardial fat on the posterior wall can also contribute to AF in two ways. Firstly, adipose tissue produces inflammatory signals that support remodelling and fibrosis [31]. Secondly, animal studies have suggested infiltration of epicardial adipose tissue into the myocardium may create tissue disorganisation that can serve as a substrate for aberrant conduction [32]. Areas of abnormal conduction in the posterior left atrium have been shown to be associated with adjacent epicardial adipose tissue in obese patients with AF [33].

### 5.3. Difficulties with Endocardial Ablation of Posterior Wall

Given the evidence for the posterior wall as an AF substrate, both in triggering and sustaining AF, the posterior wall has been explored as a target of radiofrequency and cryoablation to improve clinical outcomes in AF, particularly PersAF and LSPersAF. This is evident from the Cox-Maze IV surgical ablation lesion set, which isolates the posterior wall of the left atrium with epicardial ablation lines on the right and left pulmonary vein antrum followed by roof and floor ablations anchored to the left atriotomy [34]. However, Cox-Maze IV is typically performed concomitantly with open cardiac surgeries, limiting its reach to patients who do not need or want an open procedure.

Endocardial catheter isolation of the left atrial posterior wall has been studied with both radiofrequency and cryothermal ablation (Table 1). The majority of these studies included only patients with PersAF and LSPersAF, which is in line with current guideline recommendations when considering posterior wall isolation in conjunction with PVI [1]. Meta-analyses of a few randomised and observational comparison studies have suggested an overall benefit of endocardial posterior wall ablation compared to pulmonary vein isolation alone in PersAF [35,36], but results of the individual studies, including the randomised clinical trials [37,38,39], are mixed (Table 1). This may, in part, be due to the lack of a standardised approach to posterior wall isolation, which is evidenced by the various lesion sets used in published studies. These approaches to posterior wall isolation include a single ring around the PVs and posterior left atrium [39], linear lesions (left atrial roof and posterior-inferior) to create the so-called posterior ‘box’ lesion [37,38,40,41], or extensive point-by-point radiofrequency [42] or segmental cryoballoon ablation [43,44] to debulk the posterior wall. Adjunctive lesions also vary among these studies.

In addition to a lack of standardised posterior wall ablation strategy, other practical challenges limit the extent to which endocardial posterior wall isolation can be achieved and thus may contribute to varied clinical outcomes. One major concern with endocardial catheter ablation of the left atrial posterior wall is potential collateral damage. The tissue of the posterior wall is thin, particularly at the superior aspect, in part to accommodate the stress of limited cardiac motion at the pericardial reflections [45]. It has been shown using post-mortem hearts that the posterior wall tissue is generally thinner in patients with AF, with an overall mean thickness of ≤3 mm [45]. Endocardial catheters apply ablative energy away from the heart towards the pericardium, therefore there are risks of cardiac perforation and tamponade as well as thermal injury to the oesophagus and other adjacent structures. Atrio-oesophageal fistula is the most devastating consequence of oesophageal thermal injury. While the documented incidence is low (<0.1%) with endocardial posterior wall ablation, the potential risk remains, and the consequences can be fatal [46]. Oesophageal temperature monitoring during ablation may be used as an alert for thermal injury; however, there are well recognised limitations such as the temperature can continue to rise after ablation is stopped and the probe may cause oesophageal damage by thermal effect. Consequently, despite the use of this device, atrio-oesophageal fistula can still develop [47], limiting the widespread use of such an approach for monitoring. Indeed, a recent randomised study demonstrated a similar rate of endoscopically-detected oesophageal lesions following endocardial catheter ablation with and without the use of an oesophageal temperature probe [48]. Additionally, aborting ablation due to an unexpected rise in temperature may result in incomplete ablation lines and gaps. Reducing the power and/or duration of energy delivery during ablation on the posterior wall is normally undertaken to reduce the risk of collateral damage, but this also reduces the efficacy of lesion formation. Taken together, active mitigation of thermal injury is important, yet it may also contribute to incomplete isolation of the posterior wall and varied clinical outcomes.

Reported rates of acute and continued isolation of the posterior wall using endocardial catheter ablation suggest there is difficulty in creating transmural and durable lesions. A meta-analysis of endocardial posterior wall isolation found an acute procedural success rate of 78% (95% CI, 59.4–94.4%) with results from box, single ring and debulking techniques combined [35]. The same meta-analysis also reported a substantial rate of posterior wall reconnections observed at repeat electrophysiology procedures for arrhythmia recurrence after endocardial catheter ablation: the pooled rate of posterior wall reconnection was 63.1% (95% CI, 42.5–82.4%) [35]. Markman et al. assessed chronic posterior wall isolation at repeat ablation after a single procedure of PVI and posterior wall ablation. They found a 40% rate of posterior wall reconnections in patients who experienced arrhythmia recurrence, with most reconnections at the atrial roof and most recurrences classified as atrial flutter in patients with failed posterior wall isolation [49]. Bai et al. reported 37.5% of patients had posterior wall reconnections three months after a single endocardial posterior wall debulking procedure [42]. In fact, four of the studies comparing PVI to PVI with posterior wall isolation discussed herein suggest suboptimal durability of posterior wall isolation using endocardial catheter ablation (Table 2).

Evidence of endocardial–epicardial dissociation in atrial fibrillation may also limit the effectiveness of endocardial posterior wall isolation, especially when considered in the context of suboptimal transmurality. Endocardial–epicardial dissociation, as evidenced by asynchronous activation of the epicardial and endocardial surfaces, was initially demonstrated in animal [50] and computational models [51]. More recently, real-time mapping has shown there may be up to 50–55% asynchronous activation between the epicardial and endocardial surfaces in patients with AF [52,53]. One contributing factor to endocardial–epicardial dissociation in AF may be the presence of fibrosis in the epicardial layer, which was first suggested by animal studies [54] and recently supported by computational modelling with validation in a small number of patients [55]. The cumulative evidence for endocardial–epicardial dissociation suggests that endocardial-only mapping and ablation may be insufficient to adequately address conduction abnormalities on both cardiac surfaces in AF.

### 5.4. Hybrid Epicardial–Endocardial Approach to Address Posterior Wall Silencing

In effect, there are three main challenges in the treatment of PersAF and LSPersAF: (i) limited candidates for concomitant surgical ablation; (ii) limited effectiveness of catheter ablation in non-paroxysmal AF; (iii) challenges with endocardial catheter ablation focused on the left atrial posterior wall, which is a source of AF triggers and a substrate. These issues prompted the development of hybrid epicardial–endocardial approaches to ablation. Hybrid approaches combine minimally invasive epicardial ablation by a cardiothoracic surgeon and endocardial ablation by an electrophysiologist to complete a transmural lesion set that effectively isolates the pulmonary veins and left atrial posterior wall.

There are two general strategies for hybrid epicardial–endocardial ablation. The primary difference is the surgical epicardial ablation technique, including epicardial access, ablation tools and posterior wall lesion set. Hybrid ablation can be achieved with totally thoracoscopic (TT) epicardial ablation followed by endocardial ablation. In hybrid TT ablation, surgical access to the pericardium is achieved thoracoscopically and the epicardial lesion set is focused on PVI and creating a box lesion set across the posterior wall. Endocardial ablation is performed by an electrophysiologist to complete PVI and address gaps. The first report of this approach was published in 2011 [56]. Recent retrospective studies have reported mid-term (2–3 year) outcomes ranging from 67–79% arrhythmia-free survival off AADs in patients with PersAF and LSPersAF [57,58,59]. Safety and effectiveness of hybrid TT ablation are being evaluated in two randomised clinical trials (NCT02441738, NCT02695277) and one single-arm trial (NCT02393885).

In the other hybrid epicardial–endocardial approach, commonly referred to as the hybrid Convergent procedure, the surgeon uses a single, small subxiphoid incision to gain access to the pericardial space without the use of additional ports. It was initially proposed in 2009 [60] and the ablation set has evolved over time. In early studies, an ex-Maze lesion set was performed through a transabdominal, transdiaphragmatic approach [60]. A box lesion set then became the preferred method to isolate the posterior wall. Since 2012, epicardial posterior wall homogenization has been achieved with 2–3 rows of linear lesions spanning between the pulmonary veins [61], which is another distinction from the TT lesion set. Beginning in 2016, the pericardial space has been accessed via the subxiphoid incision [62], eliminating the need to divide the central tendon of the diaphragm. Endocardial mapping and ablation are subsequently performed by the electrophysiologist on the same day, sequential day, or several weeks later, with the goal of ensuring PVI and addressing any gaps following the epicardial procedure. Further, since there is recovery of electrical conduction following epicardial ablation, it remains important to undertake both components of the hybrid technique to achieve long-lasting, widespread transmurality [63]. Observational clinical outcomes from contemporary analyses have suggested favourable outcomes with this technique [64,65,66,67,68], which were recently corroborated by the results of the multi-centre, randomised controlled CONVERGE trial [69]. The trial compared hybrid Convergent ablation with endocardial catheter ablation in PersAF and LSPersAF and met its primary safety and effectiveness endpoints. Twelve-month freedom from atrial arrhythmias without new/increased doses of AADs was 67.7% with hybrid Convergent ablation compared to 50.0% with catheter ablation (*p* = 0.036). Significantly better effectiveness off AADs (53.5% vs. 32.0%, *p* = 0.013) and irrespective of AADs (76.8% vs. 60.0%, *p* = 0.033) were also achieved with hybrid Convergent ablation. The 30-day major adverse event rate with the hybrid Convergent procedure was 7.8% (vs 0.0% in the catheter arm, *p* = 0.0525), primarily relating to inflammatory pericardial effusions. Of note, no cardiac perforations, deaths or atrio-oesophageal fistulas occurred.

One important aspect of both hybrid ablation strategies is a collaborative, heart team approach to patient management in order to optimise clinical outcomes and safely mitigate risks [70].

## 6. Future Directions

Isolation of the left atrial posterior wall with a combination of epicardial and endocardial ablation to increase the likelihood of durable, transmural lesion has shown promising results in observational studies during the last decade as well as in a randomised controlled trial. More recently, these studies have described outcomes using a subxiphoid approach to reach the posterior left atrium, and additional studies dedicated to this approach will be important. Concomitant application of the AtriClip^®^, the most widely employed left atrial appendage exclusion device, is gaining popularity [4] and future studies should assess the precise impact on AF outcomes of including this technique. Another endpoint of interest for a hybrid approach is evaluating the length of stay for comparison with other minimally invasive surgical ablation approaches. For example, in our experience, we have seen rapid recovery after hybrid Convergent ablation, with a median length stay of 1 day, in contrast to recovery times for patients who undergo totally thoracoscopic Maze procedures, who typically require several days prior to discharge.

## 7. Conclusions

The left atrial posterior wall is likely an important driver and substrate as AF progresses and, as such, its isolation has been explored during AF ablation procedures to improve clinical outcomes in PersAF and LSPersAF. Surgical-only approaches to isolate the posterior wall are limited by invasiveness and patient eligibility for a concomitant procedure. Endocardial ablation alone to isolate the posterior wall has yielded mixed results in PersAF and LSPersAF. Electrophysiological differences between the endocardium and epicardium may not be safely addressable with an endocardial approach alone. The combination of the two concepts into a hybrid electrophysiological–surgical collaboration, such as in the Convergent procedure, may help to optimise lesion durability and transmurality to effectively isolate the posterior wall.

## Figures and Tables

**Table 1:** Summary of select studies evaluating addition of posterior wall isolation to pulmonary vein isolation.

Study	AF Type	Primary Energy Source	Ablation Strategies		Summarised Outcomes
			PVI Group	PVI + PW Group	
Aryana et al. 2018 Non-randomised	PersAF	Cryoballoon	PVI >50% received CTI by irrigated RF	PVI Segmental LAPW ablation >50% received CTI by irrigated RF Point-by-point RF as needed if isolation not achieved 32.4% had adjunct RF to complete PVI + PW	1-year freedom from atrial arrhythmias was 74% in PVI + PW vs. 48% in PVI (*p* < 0.001) ^1^
Bai et al. 2016 Non-randomised	PersAF	Irrigated RF	PVAI SVC ablation if needed Verification of PVI at 3 months and reablation if needed until isolated	PVAI to the coronary sinus and left side of interatrial septum Extensive PW ablation SVC ablation if needed Verification of PVI + PWI at 3 months and reablation if needed	1-year freedom from atrial arrhythmia off AADs was 65% in PVI + PW group vs. 20% in PVI group (*p* < 0.001); benefit maintained through 3 years
Tokioka et al. 2020 Non-randomised	PersAF	Irrigated RF	Circumferential PVI PV carina	Circumferential PVI PV carina Roof line Inferior line	AF recurrence rate was 31.1% in PVI + PW vs. 47.3% in PVI at median 19 mths (*p* = 0.35) Recurrence of PersAF was 5.6% vs. 20.9% (*p* = 0.002); no significant differences in recurrent PAF or atrial tachycardia
Tamborero et al. 2009 Randomised	60% PAF, 20% PersAF, 20% LSPAF	Irrigated RF	Circumferential PVAI MTI Roof line	Circumferential PVAI MTI Roof line Inferior line	Freedom from atrial arrhythmias at mean 9.8 mths follow-up was 55% in each group (*p* = 0.943) No significant difference in outcomes between ablation strategies in Pers/LSPersAF subanalysis
Lim et al. 2012; Randomised	61% PAF, 22% PersAF; 17% LSPAF	RF	PVAI Roof line MTI (54%) CTI	Single ring isolation MTI (54%) CTI	2-year AF-free survival was 74% in PVI + PW vs. 61% in PVI (*p* = 0.031) 2-year atrial arrhythmia-free survival was not significantly different
Kim et al. 2015 Randomised	PersAF	Irrigated RF	Circumferential PVI Roof line Anterior wall line CTI	Circumferential PVI Roof line Anterior wall line CTI Inferior line	12-month cumulative recurrence was 16.7% in PVI + PW vs. 36.7% for PVI alone (*p* = 0.02)
Lee et al. 2019 Randomised	26.7% PersAF 73.3% LSPersAF	Irrigated RF	Circumferential PVI CTI	Circumferential PVI CTI Roof line Inferior line Point ablation as needed Anterior line as per physician discretion	Freedom from AF off AADs (mean 16.2 mths) was 55.9% in PVI + PW vs. 50.5% in PVI (*p* = 0.522) Recurrence rate was 26.5% in PVI + PW vs. 23.8% in PVI (*p* = 0.78).
Aryana et al. 2021 Randomised	65.5% PersAF; 34.5% LSPAF	Cryoballoon	PVI CTI by RF Point-by-point RF as needed for PVI 7.3% had adjunct RF ablation	PVI Segmental LAPW ablation CTI by RF Point-by-point RF as needed for PVI + PW 45.5% had adjunct RF ablation	12-month AF recurrence was 25.5% in PVI + PW vs. 45.5% in PVI (*p* = 0.028) 12-month atrial arrhythmia recurrence was 34.5% in PVI + PW vs. 49.1% in PVI (*p* = 0.12)

^1^ Percentages depicted in Kaplan–Meier curve in Aryana et al. 2018 as noted in Della Rocca et al. 2020; AAD: antiarrhythmic drugs; AF: atrial fibrillation; CTI: cavotriscupid isthmus; LSPersAF: long-standing persistent AF; MTI: mitral isthmus; PAF: paroxysmal AF; PersAF: persistent AF; PVAI: pulmonary vein antrum isolation; PVI: pulmonary vein isolation; PW: posterior wall; RF: radiofrequency; SVC: superior vena cava.

**Table 2:** Posterior wall (PW) connection rates in studies comparing pulmonary vein isolation (PVI) to PVI + PW isolation.

Study	Posterior Wall Strategy	Follow-Up Time	Population Evaluated for Reconnection	Reconnection Rates in PW Ablation Group
Bai et al. 2016	Debulking with RF	3-months	All patients	37.5% ^1^
Lee et al. 2015	Linear ablation with RF	16.2 ± 8.8 months	Recurrent patients	50%
Tamborero et al. 2009	Linear ablation with RF	9.8 ± 4.3 months	Recurrent patients	67%
Tokioka et al. 2020	Linear ablation with RF	1–6 months	Recurrent patients	65.2%

^1^ Includes pulmonary vein and PW reconnections; PVI: pulmonary vein isolation; PW: posterior wall; RF: radiofrequency.
